# Cerebellar metastasis of ovarian cancer: a case report

**DOI:** 10.1186/s13256-023-04211-6

**Published:** 2023-12-19

**Authors:** Eleonora Cabitza, Marta Pirola, Cinzia Baldessari, Giuditta Bernardelli, Elena Zunarelli, Stefania Pipitone, Maria Giuseppa Vitale, Cecilia Nasso, Eleonora Molinaro, Marco Oltrecolli, Elisa D’Agostino, Vincenzo Dario Mandato, Andrea Palicelli, Massimo Dominici, Roberto Sabbatini

**Affiliations:** 1 Oncology, Azienda Ospedaliero Universitaria di Modena, Modena, Italy; 2 Pathology, Azienda USL–IRCCS di Reggio Emilia, Reggio Emilia, Italy; 3 Anatomic Pathology Unit, Azienda Ospedaliero Universitaria di Modena, Modena, Italy; 4 Oncology, Ospedale S. Corona, Pietra Ligure, Italy; 5 Oncology, AUSL Modena – Distretto di Sassuolo, Sassuolo, MO Italy; 6 Gynaecology, Azienda USL-IRCCS di Reggio Emilia, Reggio Emilia, Italy

**Keywords:** Ovarian cancer, Brain metastasis, PARP inhibitor, Multimodal approach, Case report

## Abstract

**Background:**

Ovarian cancer is metastatic at presentation in about 62% of cases, but brain metastases are rare, reported in 3.3–4% of patients. Brain metastasis seems to be more frequent in advanced stages at diagnosis and in patients with *BRCA1/2* mutation.

**Case presentation:**

We present a case of a 47-year-old Caucasian woman, *BRCA *wild type, with an ovarian cancer that started with single cerebellar metastasis.

**Conclusion:**

Brain metastases in ovarian cancer are rare and complex for diagnosis and management. This case focuses both on diagnosis and treatment, emphasizing the importance of a multimodal approach in a multidisciplinary team.

## Background

Ovarian cancer (OC) is the deadliest gynecological tumor, representing the fifth cause of death from cancer among women, as 7.2 deaths per 100,000 women are reported every year in the USA [1]. OC is a “silent killer,” being metastatic at presentation in about 62% of cases; indeed, its diagnosis often occurs in advanced stage (FIGO stage III–IV) [1, 2].

The most common sites of metastasis are the peritoneum, liver, and lymph nodes [3, 4]. Only occasionally, bones could be involved [3–5]. Conversely, brain metastases (BMs) are rare; they are globally reported in 3.3–4% of patients, as confirmed by the Multicenter Italian Trials in Ovarian cancer (MITO) analysis [5, 6]. A progressive increase in BMs incidence has been observed, probably due to the improvement in diagnostic techniques and in patients’ survival with new therapeutic options [7, 8]. The identification of BMs at presentation is still exceptional (only 1% of patients), as emerged from the retrospective study of Gardner *et al.* [9]. BMs seem to be more frequent in patients with OC with poorly differentiated histology (serous, clear cell, undifferentiated) and advanced stage at diagnosis, but they may occur even in the IC stage [6, 10]. It is reported that patients with OC with a *BRCA1/2* mutation are more prone to develop both visceral and BMs compared with *BRCA* wild-type patients [11–14]. As emerged from the retrospective study of Teckie *et al.*, most patients presenting with encephalic metastasis have a single metastasis (about 46.7%) while 26.7% have four metastases or more; leptomeningeal disease is still rarely reported in ovarian cancer [14]. BMs are related with poor prognosis [5, 13], and, regardless of *BRCA* status, patients with OC with lung, bone, or brain metastases have a worse prognosis compared with those who have liver metastases [15].

The current standard treatment of epithelial ovarian cancer (EOC) of all histological subtypes involves primary optimal debulking surgery followed by cisplatin-based chemotherapy. Metastasis to the central nervous system (CNS) from OC has been postulated to occur via direct hematogenous seeding through Virchow–Robin perivascular spaces, through retrograde lymphatic spread in the case of meningeal involvement, or by direct invasion into the central nervous system after bony involvement [16].

The management of BMs requires a multimodal approach [10, 16]. Surgery associated with chemotherapy and/or radiotherapy could ensure not only a better effective response, but also a longer survival [10, 16–19].

Herein we report on the case of a patient suffering from high-grade serous ovarian carcinoma whose symptoms at presentation and initial diagnosis were related with a cerebellar metastasis. The metastasis and the primary tumor were surgically treated. Subsequently, the patient underwent adjuvant chemotherapy and maintenance with PARP inhibitor (PARPi) niraparib.

## Case presentation

On 13 September 2021, a young Caucasian woman under 50 years old presented to the emergency department with a headache that had been recurring for about a month and worsening. The patient did not have side deficits or other neurological disorders. Physical examination showed no major symptoms, and blood tests performed in an emergency revealed no criticality. She did not describe weight loss or other symptoms suggesting cancer.

**Fig. 1:**
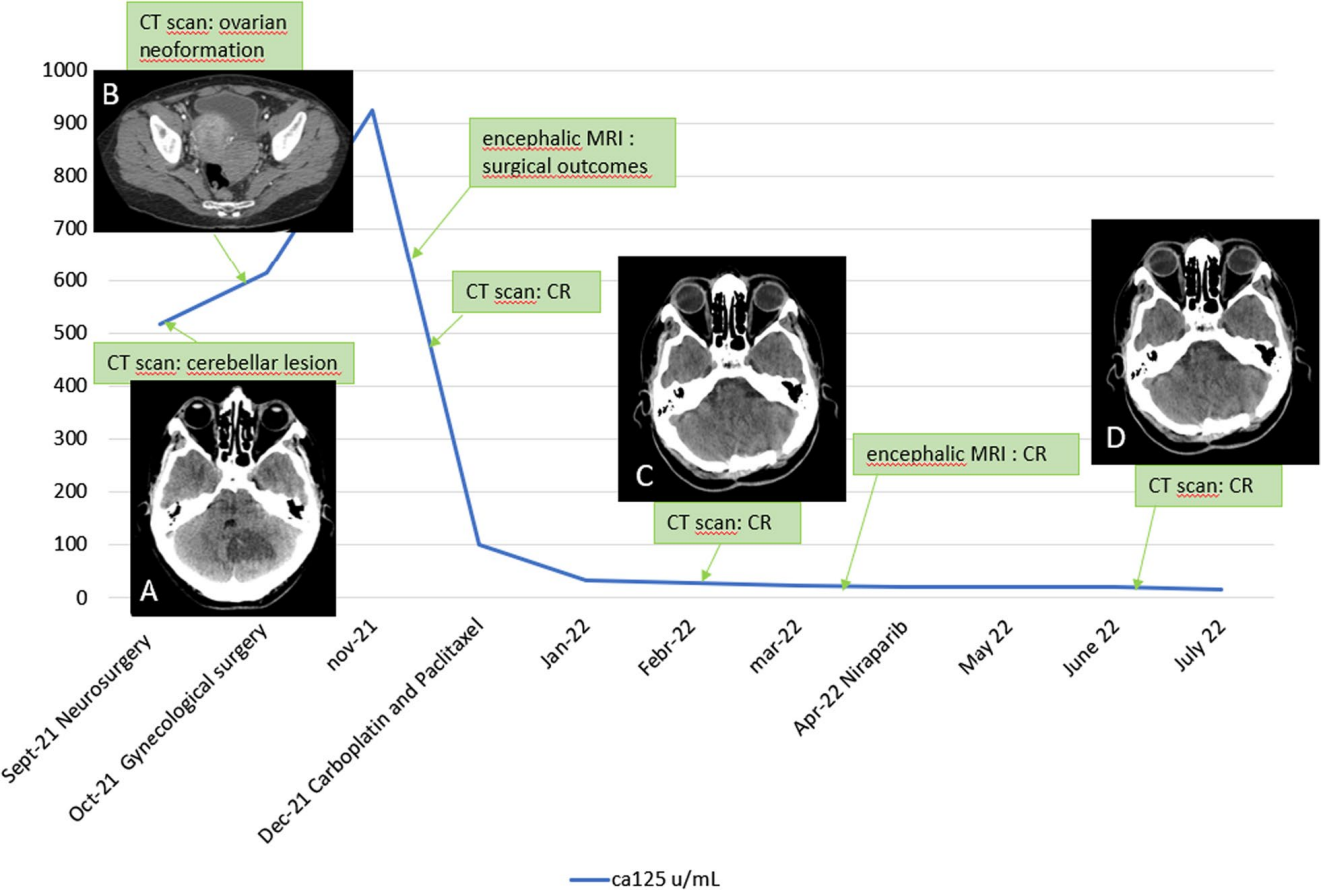
CA125 Marker trend. **A** Brain CT with cerebellar lesion. **B** Abdominal CT with ovarian neoformation. **C** Brain CT 5 months after surgery. **D** Brain CT 9 months after surgery

Brain computed tomography (CT) scans revealed the presence of a left cerebellar voluminous cystic lesion, with mass effect and obstructive hydrocephalus (Fig. 1). She was an active smoker and her medical history included hypertension, hypercholesterolemia under treatment, gastroesophageal reflux disease, and an episode of non-ST-segment elevation myocardial infarction (NSTEMI) treated with stent placement in July 2020. She did not report major surgical interventions and denied family history of oncological pathologies.

On 16 September 2021, she underwent neurosurgery for the removal of the cerebellar lesion.

**Fig. 2:**
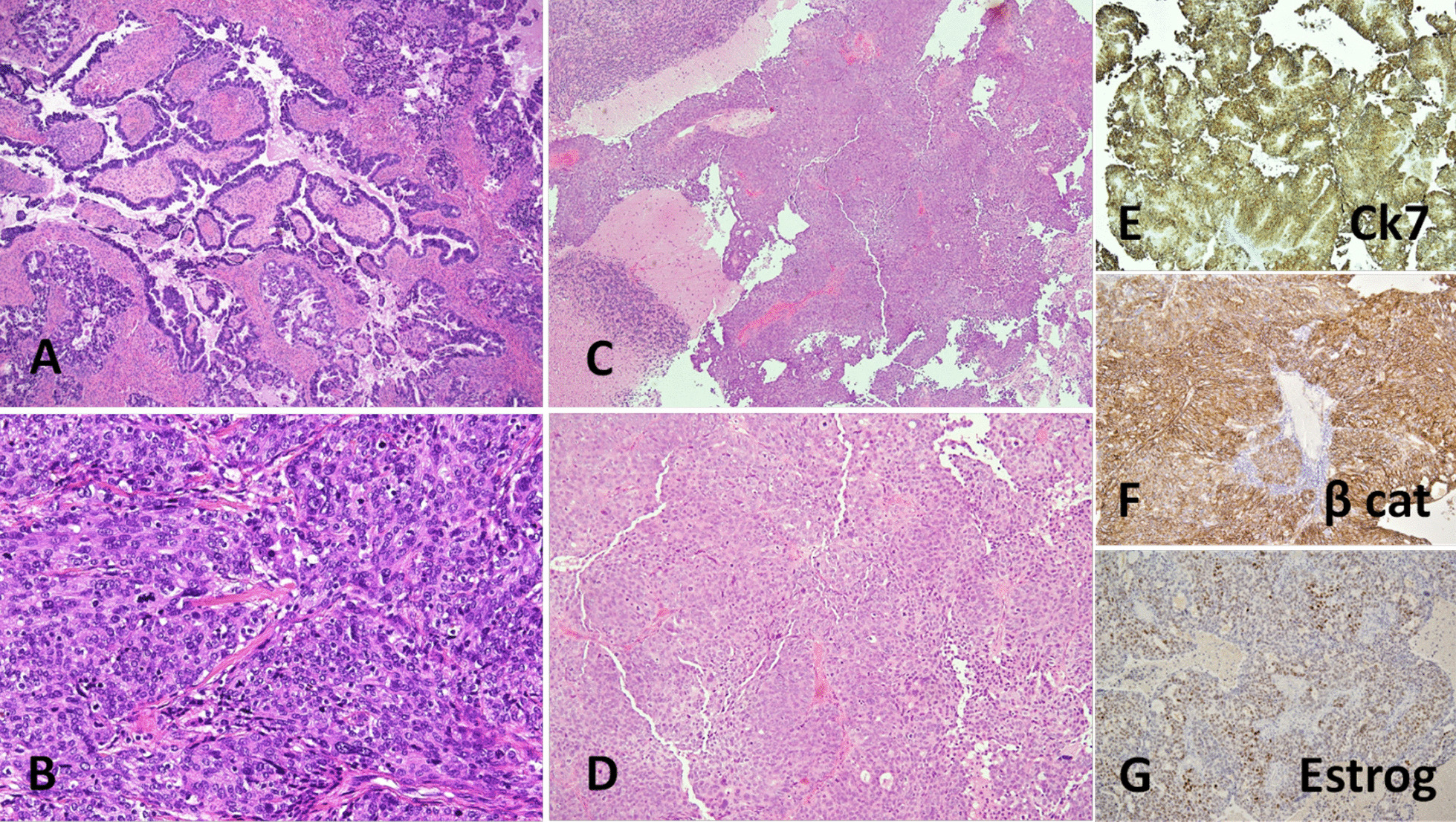
Microphotographs of the ovary tumor (**A**, **B**) and cerebellar lesion (**C**, **D**), hematoxylin–eosin stains; 4× magnification (**A**, **C**) and 10× magnification (**B**, **D**). **A** Glandular and papillary patterns of high-grade serous carcinoma with significant nuclear pleomorphism. **B** Solid appearance of high-grade serous carcinoma with significant nuclear atypia, eosinophilic cytoplasm and high mitotic index. **C**, **D** Poorly differentiated adenocarcinoma with papillary aspects. **E**, **F** Immunohistochemical stains of cerebellar lesion, witch suggested the secondary origin of the lesion from an ovarian primary: cytokeratin 7 (CK7) (**E**), β-catenin (β-cat) (**F**), and estrogen (Estrog) (**G**)

On histopathological examination, a poorly differentiated adenocarcinoma with papillary aspects was found (Fig. 2C, D). Morphology and immunophenotype (CK7+, ER+/−, PR−/+, CD10−/+, c-ERBB2−, TTF1−, CDX-2−, CK20−, β-catenin−) were consistent with a metastasis from an ovarian/gynecological primitivity (Fig. 2E–G).

Contrast-enhanced chest and abdominal computed tomography (CT) scans reported an oval-shaped neoformation of 12.9 × 7.7 cm with bumpy contours located in the right adnexal area, which was suspicious for ovarian carcinoma; an oval-shaped solid formation of 5.8 × 5.6 × 3.7 cm was also found in the left adnexal area, while no other pathological findings were documented elsewhere (Fig. 1). The lesions were confirmed with magnetic resonance imaging (MRI) and transvaginal gynecologic ultrasound scans. In September 2021, serum tumor markers were carcinoembryonic antigen (CEA) 5.8 ng/mL [normal value (n.v.) 0–5 ng/mL] and cancer antigen 125 (CA125) 614.6 U/mL (n.v. 0–35 U/mL).

In October 2021, she underwent laparoscopic bilateral adnexectomy and peritoneal washing cytology. On histopathological examination, the peritoneal fluid was negative for neoplastic cells. In both ovaries there was evidence of high-grade, infiltrating serous carcinomas, with lymphovascular invasion and extension to the serosa (Fig. 2A and B). The final stage was pT1c2 cN0 pM1b (FIGO stage IV). No *BRCA* mutations were detected. On 2 November 2021, the postsurgical chest and abdominal CT revealed no evidence of residual disease.

On 8 November 2021, the brain MRI showed in the left deep cerebellar hemisphere—the site of surgery—an area with fluid content and thickened margins avidly enhancing after contrast administration. At this time, serum tumor markers were CEA 5.4 ng/mL and CA 125 99.8 U/mL. The case was discussed in a multidisciplinary meeting with no indication for brain radiotherapy (RT).

From December 2021 to April 2022, the patient underwent first-line chemotherapy with carboplatin and paclitaxel three-weekly administrations, standard doses, that was well tolerated in the absence of significant side effects. On 23 December 2021, about 2.5 months after surgery, serum tumor markers were all in the range of normality.

In February 2022, the multidisciplinary team placed the indication to repeat brain MRI with the aim of evaluating whether to perform brain stereotactic RT. In the meantime, bevacizumab was not added to chemotherapy. Thoracic and abdominal CT scan performed on 24 February 2022 confirmed no evidence of residual disease. On 17 March 2022, the brain MRI showed findings consistent with postsurgical sequelae and RT was not confirmed.

On March 2022, the patient ended chemotherapy after six administrations with persistence of the complete response obtained from surgery. On 28 April 2022, she started maintenance therapy with PARPi niraparib 200 mg daily, representing the standard schedule and dose for her weight. The first CT scan after starting the maintenance therapy (June 2022) confirmed complete response. On 21 July 2022, the serum CEA levels were 5.4 ng/mL while CA125 levels accounted for 16.8 U/mL. Brain MRI, repeated in April 2022 and in July 2022, were negative for disease. The patient is alive and continues maintenance treatment with good quality of life at the time of writing.

## Discussion and conclusion

BMs in ovarian cancer are rare but complex for diagnosis and management as they impact the prognosis of patients. This case focuses both on diagnosis and treatment, emphasizing the importance of a multimodal approach in a multidisciplinary team.

In this case, the presentation with a cerebellar metastasis of ovarian carcinoma at diagnosis in exceptionally rare. The diagnosis was based on morphological and immunohistochemical data, confirmed by clinical and radiological findings.

Furthermore, even in the absence of a consensus-based treatment protocol, the multimodal therapeutic strategy is showing excellent results, with surgery upfront and systemic therapy later. Among the maintenance strategies for OC, the PARPi niraparib has already demonstrated its efficacy in the treatment of BMs in animal models, being able to overcome the blood–brain-barrier [20]. The case of a patient on maintenance therapy with niraparib who was in remission for over 17 months with an excellent quality of life was reported [21].

This case represents an example of successful personalized multimodal diagnostic and therapeutic approach in an atypical presentation of stage IV ovarian cancer with a cerebellar metastasis at diagnosis, that has warranted favorable results in the control of onset symptoms and metastatic disease.

## Data Availability

The original contributions presented in the study are included in the article, further inquiries can be directed to the corresponding author/s.

## Abbreviations

BMs: Brain metastases
CA125: Cancer antigen 125
CEA: Carcinoembryonic antigen
CT: Computed tomography
FIGO: International Federation of Gynecology and Obstetrics
MITO: Multicenter Italian Trials in Ovarian cancer
MRI: Magnetic resonance imaging
OC: Ovarian cancer
PARPi: PARP inhibitor

## References

[1] Jelovac D, Armstrong DK (2011). Recent progress in the diagnosis and treatment of ovarian cancer. CA Cancer J Clin.

[2] Torre LA, Bray F, Siegel RL, Ferlay J, Lortet-Tieulent J, Jemal A (2015). Global cancer statistics, 2012. CA Cancer J Clin.

[3] Rose PG, Piver MS, Tsukada Y, Lau TS (1989). Metastatic patterns in hisologic variants of ovarian cancer. An autopsy study. Cancer.

[4] Güth U, Huang DJ, Bauer G, Stieger M, Wight E, Singer G (2007). Metastatic patterns at autopsy in patients with ovarian carcinoma. Cancer.

[5] Marchetti C, Ferrandina G, Cormio G, Gambino A, Cecere S, Lorusso D (2016). Brain metastases in patients with EOC: Clinico-pathological and prognostic factors. A multicentric retrospective analysis from the MITO group (MITO 19). Gynecol Oncol.

[6] Geisler JP, Geisler HE (1995). Brain metastases in epithelial ovarian carcinoma. Gynecol Oncol.

[7] Piura E, Piura B (2011). Brain metastases from ovarian carcinoma. ISRN Oncol.

[8] Kolomainen DF, Larkin JM, Badran M, A'Hern RP, King DM, Fisher C, Bridges JE, Blake PR, Barton DP, Shepherd JH, Kaye SB, Gore ME (2002). Epithelial ovarian cancer metastasizing to the brain: a late manifestation of the disease with an increasing incidence. J Clin Oncol.

[9] Gardner AB, Charo LM, Mann AK, Kapp DS, Eskander RN, Chan JK (2020). Ovarian, uterine, and cervical cancer patients with distant metastases at diagnosis: most common locations and outcomes. Clin Exp Metastasis.

[10] McMeekin DS, Kamelle SA, Vasilev SA, Tillmanns TD, Gould NS, Scribner DR, Gold MA, Guruswamy S, Mannel RS (2001). Ovarian cancer metastatic to the brain: what is the optimal management?. J Surg Oncol.

[11] Gourley C, Michie CO, Roxburgh P, Yap TA, Harden S, Paul J, Ragupathy K, Todd R, Petty R, Reed N, Hayward RL, Mitchell P, Rye T, Schellens JH, Lubinski J, Carmichael J, Kaye SB, Mackean M, Ferguson M (2010). Increased incidence of visceral metastases in Scottish patients with BRCA1/2-defective ovarian cancer: an extension of the ovarian BRCAness phenotype. J Clin Oncol.

[12] Ratner E, Bala M, Louie-Gao M, Hazard S, Brastianos P (2018). Brain metastases in primary ovarian cancer: real-world data. Ann Oncol.

[13] Jernigan AM, Mahdi H, Rose PG (2015). Epithelial ovarian cancer metastatic to the central nervous system and a family history concerning for hereditary breast and ovarian cancer–a potential relationship. Int J Gynecol Cancer.

[14] Teckie S, Makker V, Tabar V, Alektiar K, Aghajanian C, Hensley M, Beal K (2013). Radiation therapy for epithelial ovarian cancer brain metastases: clinical outcomes and predictors of survival. Radiat Oncol.

[15] Deng K, Yang C, Tan Q, Song W, Lu M, Zhao W, Lou G, Li Z, Li K, Hou Y (2018). Sites of distant metastases and overall survival in ovarian cancer: a study of 1481 patients. Gynecol Oncol.

[16] Pectasides D, Pectasides M, Economopoulos T (2006). Brain metastases from epithelial ovarian cancer: a review of the literature. Oncologist.

[17] Anupol N, Ghamande S, Odunsi K, Driscoll D, Lele S (2002). Evaluation of prognostic factors and treatment modalities in ovarian cancer patients with brain metastases. Gynecol Oncol.

[18] Cormio G, Loizzi V, Falagario M, Lissoni AA, Resta L, Selvaggi LE (2011). Changes in the management and outcome of central nervous system involvement from ovarian cancer since 1994. Int J Gynaecol Obstet.

[19] Growdon WB, Lopez-Varela E, Littell R, Oliva E, Seiden M, Krasner C, Lee H, Fuller A (2008). Extent of extracranial disease is a powerful predictor of survival in patients with brain metastases from gynecological cancer. Int J Gynecol Cancer.

[20] Mikule K, Wilcoxen K. The PARP inhibitor, niraparib, crosses the blood brain barrier in rodents and is efficacious in a BRCA2-mutant intracranial tumor model [abstract]. In: Proceedings of the AACR-NCI-EORTC International Conference: Molecular Targets and Cancer Therapeutics; 2015 Nov 5–9; Boston, MA. Philadelphia (PA): AACR; Mol Cancer Ther 2015;14(12 Suppl 2):Abstract nr B168_._

[21] Gray S, Khor XY, Yiannakis D (2019). Niraparib as maintenance therapy in a patient with ovarian cancer and brain metastases. BMJ Case Rep.

